# The role of interoception in the mechanism of pain and fatigue in fibromyalgia and myalgic encephalomyelitis/chronic fatigue syndrome (ME/CFS)

**DOI:** 10.1192/j.eurpsy.2021.382

**Published:** 2021-08-13

**Authors:** H. Sharp, K. Themelis, M. Amato, A. Barritt, K. Davies, N. Harrison, H. Critchley, S. Garfinkel, J. Eccles

**Affiliations:** 1 Department Of Neuroscience, Brighton and Sussex Medical School, PX, United Kingdom; 2 Psychiatry, Sussex Partnership NHS Foundation Trust, EP, United Kingdom; 3 Department Of Psychology, University of Warwick, AL, United Kingdom; 4 Department Of Clinical And Experimental Medicine, Brighton and Sussex Medical School, PX, United Kingdom; 5 Cardiff University Brain Research Imaging Centre, University of Cardiff, HQ, United Kingdom; 6 Institute Of Cognitive Neuroscience, University College London, AZ, United Kingdom

**Keywords:** Interoception, Pain, fatigue, Anxiety

## Abstract

**Introduction:**

Pain, fatigue and anxiety are common features of fibromyalgia and ME/CFS and significantly impact quality of life. Aetiology is poorly defined but dysfunctional inflammatory, autonomic and interoceptive (sensing of internal bodily signals) processes are implicated.

**Objectives:**

To investigate how altered interoception relates to baseline expression of pain, fatigue and anxiety symptoms in fibromyalgia and ME/CFS and in response to an inflammatory challenge.

**Methods:**

Sixty-five patients with fibromyalgia and/or ME/CFS diagnosis and 26 matched controls underwent baseline assessment: pressure-pain thresholds and self-report questionnaires assessing pain, fatigue and anxiety severity. Participants received injections of typhoid (inflammatory challenge) or saline (placebo) in a randomised, double-blind, crossover design, before completing heartbeat tracking tasks. Three interoception dimensions were examined: subjective sensibility, objective accuracy and metacognitive awareness. Interoceptive trait prediction error was calculated as discrepancy between accuracy and sensibility.

**Results:**

Patients with fibromyalgia and ME/CFS had significantly higher interoceptive sensibility and trait prediction error, despite no differences in interoceptive accuracy. Interoceptive sensibility and trait prediction error correlated with all self-report pain, fatigue and anxiety measures, and with lower pain thresholds. Anxiety mediated the positive-predictive relationships between pain (Visual Analogue Scale and Widespread Pain Index), fatigue impact and interoceptive sensibility. After inflammatory challenge, metacognitive awareness correlated with baseline self-reported symptom measures and lower pain thresholds.

**Conclusions:**

This is the first study investigating interoceptive dimensions in patients with fibromyalgia and ME/CFS, which were found to be dysregulated and differentially influenced by inflammatory mechanisms. Interoceptive processes may represent a new potential target for diagnostic and therapeutic investigation in these poorly understood conditions.

**Disclosure:**

No significant relationships.

